# Elevated Pulse Pressure is Associated with Hemolysis, Proteinuria and Chronic Kidney Disease in Sickle Cell Disease

**DOI:** 10.1371/journal.pone.0114309

**Published:** 2014-12-05

**Authors:** Enrico M. Novelli, Mariana Hildesheim, Caterina Rosano, Rebecca Vanderpool, Marc Simon, Gregory J. Kato, Mark T. Gladwin

**Affiliations:** 1 Vascular Medicine Institute and Division of Hematology/Oncology, University of Pittsburgh, Pittsburgh, Pennsylvania, United States of America; 2 Critical Care Medicine Department, Clinical Center, National Institutes of Health, Bethesda, Maryland, United States of America; 3 Department of Epidemiology, University of Pittsburgh, Pittsburgh, Pennsylvania, United States of America; 4 Vascular Medicine Institute and Division of Cardiology, University of Pittsburgh, Pittsburgh, Pennsylvania, United States of America; 5 Vascular Medicine Institute, Division of Pulmonary, Allergy, Critical Care Medicine, University of Pittsburgh, Pittsburgh, Pennsylvania, United States of America; Rouen University Hospital, France

## Abstract

A seeming paradox of sickle cell disease is that patients do not suffer from a high prevalence of systemic hypertension in spite of endothelial dysfunction, chronic inflammation and vasculopathy. However, some patients do develop systolic hypertension and increased pulse pressure, an increasingly recognized major cardiovascular risk factor in other populations. Hence, we hypothesized that pulse pressure, unlike other blood pressure parameters, is independently associated with markers of hemolytic anemia and cardiovascular risk in sickle cell disease. We analyzed the correlates of pulse pressure in patients (n  =  661) enrolled in a multicenter international sickle cell trial. Markers of hemolysis were analyzed as independent variables and as a previously validated hemolytic index that includes multiple variables. We found that pulse pressure, not systolic, diastolic or mean arterial pressure, independently correlated with high reticulocyte count (beta  =  2.37, p  =  0.02) and high hemolytic index (beta  =  1.53, p = 0.002) in patients with homozygous sickle cell disease in two multiple linear regression models which include the markers of hemolysis as independent variables or the hemolytic index, respectively. Pulse pressure was also independently associated with elevated serum creatinine (beta  =  3.21, p  =  0.02), and with proteinuria (beta  =  2.52, p  =  0.04). These results from the largest sickle cell disease cohort to date since the Cooperative Study of Sickle Cell Disease show that pulse pressure is independently associated with hemolysis, proteinuria and chronic kidney disease. We propose that high pulse pressure may be a risk factor for clinical complications of vascular dysfunction in sickle cell disease. Longitudinal and mechanistic studies should be conducted to confirm these hypotheses.

## Introduction

Findings from the landmark Cooperative Study of Sickle Cell Disease (CSSCD) revealed that sickle cell disease (SCD) patients, particularly those affected by HbSS, have lower systolic and diastolic blood pressure values compared to age, sex and race-matched normative values.[1] This large study confirmed findings of prior smaller series showing lower baseline blood pressure values are characteristic of SCD patients.[2], [3]

Multiple hypotheses have been formulated over the following years to explain the paradox of a population affected by severe endotheliopathy and vasculopathy and accelerated organ damage, yet largely spared from systemic hypertension. Possible explanations have taken into consideration the role of anemia, resulting in increased cardiac output with compensatory decreased vascular resistance, hyposthenuria leading to sodium loss, decreased blood viscosity of oxygenated sickle blood at low hematocrit levels, and the role of compensatory increases in cyclooxygenase-2, endothelial nitric oxide synthase, placenta growth factor and other endothelial-derived factors.[4]–[7] Again, paradoxically, endothelial function studies in humans clearly show that many patients, especially those with higher rates of hemolytic anemia, exhibit impaired response to major endothelial vasodilators, such as nitric oxide.[6], [8], [9] More recent studies have also shown that when an appropriate control population is selected and potential confounders are accounted for statistically, the blood pressure difference between SCD patients and control subjects is attenuated.[5] Adjustment for the lower BMI of SCD patients as compared to that of control subjects has proved to be particularly important in evaluating blood pressure differences.[5], [10], [11] Another possibility is that systolic, diastolic and mean arterial pressure may not be the systemic pressure parameters most reflective of SCD vasculopathy. This hypothesis is supported by recent epidemiological evidence showing that systolic blood pressure rises as biomarkers of pulmonary hypertension and intravascular hemolysis increase, while diastolic blood pressure decreases as hemoglobin levels drop in SCD, leading to subgroups of patients developing systolic hypertension and increased pulse pressure.[12]

Elevated pulse pressure has recently emerged as an independent predictor of cardiovascular outcomes and prognostic factor for death in patients at high risk of cardiovascular morbidity and mortality such as aging patients and those affected by advanced chronic kidney disease.[13], [14] The main determinants of pulse pressure are the left ventricular ejection fraction, arterial stiffness, and the timing and intensity of the backward pressure wave reflections from the periphery of the vascular system.[15] From the hemodynamic factors that influence pulse pressure, two have been shown to independently predict cardiovascular risk: aortic stiffness, measured from the aortic pulse wave velocity, and early return of reflected waves to the heart, evaluated from pulse wave analysis.[15] Under physiological conditions in young subjects, the backward pressure wave returns from the distal arterial compartment during diastole, cause pulse pressure to be higher in peripheral than in central arteries, a phenomenon known as pulse pressure amplification.[15] In conditions where pulse wave velocity and arterial stiffness are increased, such as SCD,[16] the reflected wave occurs earlier affecting the central arteries during systole. As a result of this early wave reflection, aortic and ventricular pressures are increased during systole and aortic pressure is reduced during diastole,[15] leading to increased pulse pressure. The result of increased pulse pressure is greater vascular load on the heart, which can lead to myocardial hypertrophy and heart failure,[17] and end-organ damage in other vascular districts, including the kidney (reviewed in[18]) and the brain.[19]

Hemolysis in SCD causes endothelial dysfunction via multiple mechanisms, and both hemolysis and endothelial dysfunction may be independently linked to elevated pulse pressure. High plasma levels of free hemoglobin from hemolyzed RBC lead to nitric oxide depletion and reactive oxygen species formation which cause impaired vascular relaxation and increased arterial stiffness.[20] In addition, ischemia/reperfusion injury from acute episodes of vaso-occlusion and hemolysis also lead to the generation of pathologic reactive oxygen species responsible for endotheliopathy and endothelial inflammation.[21], [22] The hemodynamic consequences of high baseline hemolysis and anemia are high cardiac output and decreased peripheral vascular resistance. The combination of increased arterial stiffness from endothelial dysfunction and a high cardiac output state from severe hemolysis and anemia are expected to elevate pulse pressure in SCD. We, therefore, hypothesized that hemolysis would be significantly associated with pulse pressure in patients with HbSS disease. In the study presented herein, we report the correlates of pulse pressure in the Treatment of Pulmonary Hypertension and Sickle Cell Disease with Sildenafil Therapy (walk-PHaSST) cohort.

## Methods

### Study Design and Selection of Subjects

The study population and design have been described in detail elsewhere.[23], [24] Overall, we recruited 720 subjects age 12 and over at steady state from nine different study sites in the United States and one site in the United Kingdom, 671 (93.2%) of whom enrolled in the study. We analyzed 661 patients with available systolic and diastolic blood pressure measurements (98.5% of enrolled subjects).

### Ethics Statement

Local institutional review boards (University of Pittsburgh IRB PRO07060076) approved the protocol and written informed consent was obtained and approved by the IRB (Clinicaltrials.gov identifier NCT00492531). This study is an analysis of data from the walk-PHaSST trial.

### Evaluation of Subjects

All screening study subjects were evaluated by histories of clinical events and lifetime treatments, physical examination, laboratory screening, transthoracic Doppler echocardiography, and the six-minute walk test. Blood pressure was obtained according to the standard method at each site with the cuff placed in the forearm and the patient seated. Routine laboratory tests including complete blood count, serum chemistry profile, lactate dehydrogenase (LDH), urinalysis, urine albumin, and urine creatinine from samples taken at the subject's screening visit were performed in the local laboratories of the participating institutions. Echocardiography was performed at the participating institutions and read centrally in the National Heart, Lung and Blood Institute echocardiography core laboratory. Cardiac measurements were performed according to American Society of Echocardiography guidelines.[25] Percentage of hemoglobin F was measured by high-performance liquid chromatography (Ultra Resolution System, Trinity Biotech, Jamestown, NY). Alpha-thalassemia was detected by molecular methodology based on polymerase chain reaction at the University of Pittsburgh. Serum N-terminal pro-brain natriuretic peptide (NT-pro BNP) concentration was measured by a sandwich immunoassay using polyclonal antibodies that recognize epitopes located in the N-terminal segment (1–76) of pro-BNP (1–108) (Elecsys analyser; Roche Diagnostics, Mannheim, Germany). Ferritin was measured with an enzyme immunoassay (Ramco Laboratories Inc., Stafford, TX). The eGFR was calculated using the CKD-EPI formula (eGFR in ml/min/1.73 m^2^ = 175 × (serum creatinine)^-1.154^ × (age)^−0.203^ × (0.742 if female) × (1.212 if African American)).

### Statistical Analysis

Patient characteristics are presented as median and interquartile range (IQR) or number and percentage of participants with a given characteristic, and associations with hemoglobin genotype were assessed using the Wilcoxon two-sample test and the Pearson chi-square test of independence. A hemolytic component variable was derived using principal component analysis from four markers of hemolysis - LDH, aspartate aminotransferase (AST), total bilirubin, and reticulocyte percent - as described elsewhere.[24] This variable produced by data reduction methodology has strong relationships with indirect and direct measures of hemolysis, including cell-free hemoglobin levels and red blood cell microparticle counts.[12] Proteinuria was defined as any positive result from the urine dipstick test from urine samples taken at the subject's screening visit. Urine albumin and creatinine were available for a subset of subjects and were measured from spot urine samples taken at the screening visit. Chronic kidney disease (CKD) was defined according to the National Kidney Foundation, Kidney Disease Outcomes Quality Initiatives (K/DOQI) guidelines (National Kidney Foundation, 2002) to align the definition of CKD stage to the current evidence-based guidelines: stage 0 – eGFR> 60 ml/min/1.73 m^2^ and albuminuria <30 mg/g creatinine; stage 1 – eGFR ≥ 90 ml/min/1.73 m^2^ and albuminuria ≥ 30 mg/g creatinine; stage 2 – eGFR 60–89 ml/min/1.73 m^2^ and albuminuria ≥ 30 mg/g creatinine; stage 3 – eGFR <60 ml/min/1.73 m^2^.

Associations of patient characteristics with pulse pressure were assessed using Spearman correlation coefficients and linear regression analysis, log-transforming continuous variables as necessary to normalize skewed distributions. Regression coefficients were tested for significant differences from zero by the t-test. For the final models, variables were entered in a stepwise approach if they had a significant univariate association with pulse pressure. All statistical analyses were performed using SAS, version 9.1 (SAS Institute, Inc., Cary, NC) and Stata, version 11.1 (Statacorp, LP, College Station, TX).

## Results

### Patient characteristics by hemoglobin genotype

Most patients enrolled in walk-PHaSST had either HbSS or HbSC and were analyzed separately. We are reporting their baseline characteristics as these are not available from prior walk-PHaSST published reports (Table 1). As expected based on prior epidemiological studies, we found significant differences among subjects with HbSS and HbSC disease. HbSS patients were significantly younger and had lower BMI and oxygen saturation than patients with HbSC disease. Among the blood pressure parameters, pulse pressure was the only one higher in the HbSS group, while systolic, diastolic and mean arterial pressures were all significantly higher in HbSC patients. The prevalence of patients on hydroxyurea was higher in the HbSS group as compared to the HbSC group, which resulted in higher fetal hemoglobin levels. There was also a higher proportion of patients on chronic transfusion in the HbSS group, which was accompanied by a higher ferritin value in this group. HbSS patients had higher markers of hemolysis as measured by reticulocyte proportion, LDH, AST, total and hemolytic component, all validated by specific measures of cell free plasma hemoglobin and red cell derived microparticles.[12] As a result of the higher hemolysis, the total hemoglobin level was lower in patients with HbSS than in patients with HbSC. A larger proportion of HbSS patients had proteinuria, a marker of sickle cell nephropathy, recently shown to be predicted by hemolysis and hemoglobinuria,[26] and chronic kidney disease stage I or higher. In keeping with these findings, they had a higher estimated glomerular filtration rate, indicative of worse glomerular hyperfiltration. Finally, the surrogate markers of pulmonary hypertension TRV and NT-proBNP were significantly different among the two genotypes.

**Table 1:** Patient characteristics by sickle cell disease genotype.

Patient characteristics	HbSS patients		HbSC patients		
	n	median (IQR)*	n	median (IQR)*	p^†^
Age, years	500	34 (25–45)	161	41 (28–51)	0.002
Male gender, N (%)	500	245 (49.0)	161	66 (41.0)	0.08
SBP, mmHg	500	117 (109–127)	161	120 (112–132)	0.01
DBP, mmHg	500	67 (60–74)	161	71 (67–80)	<0.0001
MAP, mmHg	500	83 (77–90)	161	88 (82–96)	<0.0001
Pulse pressure, mmHg	500	50 (42–59)	161	48 (41–55)	0.02
Body mass index (BMI)	492	23.0 (20.8–25.8)	160	26.7 (22.7–30.5)	<0.0001
Oxygen saturation^‡^, %	497	97 (95–98)	160	98 (97–99)	<0.0001
Hydroxyurea use, N (%)	500	225 (45.0)	161	21 (13.0)	<0.0001
Transfusions^§^, N (%)	499	72 (14.4)	159	6 (3.8)	0.0003
Chronic pain, N (%)	499	184 (36.9)	161	78 (48.4)	0.009
Six minute walk, m	492	438 (384–504)	159	440 (363–495)	0.32
WBC, × 10^9^/L	490	9.7 (7.5–4.3)	156	8.1 (6.4–10.0)	<0.0001
Hemoglobin, mmol/L	489	5.4 (4.7–6.1)	157	6.9 (6.4–7.6)	<0.0001
Hematocrit, %	490	25.2 (22.0–28.4)	157	32.4 (29.4–35.3)	<0.0001
Platelets, × 10^9^/L	489	364 (285–456)	156	296 (193–362)	<0.0001
Hemoglobin F, %	445	6.3 (2.6–12.4)	139	1.5 (0.8–3.2)	<0.0001
Reticulocytes, %	461	9.1 (6.1–13.4)	153	3.3 (2.5–4.7)	<0.0001
LDH, IU/L	453	422 (299–600)	154	240 (200–321)	<0.0001
Total Bilirubin, µmol/L	490	46.2 (32.5–70.1)	159	22.2 (15.4–34.2)	<0.0001
Hemolytic Component	420	0.6 (−0.4–1.4)	148	−1.6 (−2.3–1.0)	<0.0001
AST, IU/L	477	42 (31–59)	157	26 (21–35)	<0.0001
Ferritin, pmol/L	450	643 (254–1539)	141	263 (148–483)	<0.0001
Albumin, g/L	489	42 (39–44)	157	42 (39–44)	0.59
ALT, IU/L	491	23 (17–33)	159	18 (14–26)	<0.0001
ALP, IU/L	489	90 (70–122)	157	76 (65–102)	0.0002
Creatinine, µmol/L	492	61.9 (50.4–79.6)	159	70.7 (61.9–86.6)	<0.0001
Proteinuria, N (%)	474	162 (34.2)	147	28 (19.0)	0.0005
ACR	306	32 (7.3–220.0)	81	15.2 (4.2–68.4)	0.004
eGFR (ml/min/1.73 m^2^)	492	138 (109–153)	157	119 (95–137)	<0.0001
CKD^||^, N (%)	313	173 (55.3)	82	35 (42.7)	0.04
TRV, m/sec	449	2.6 (2.3–2.7)	139	2.4 (2.2–2.7)	0.003
NT-proBNP, pmol/L	464	9.1 (4.0–19.9)	146	5.3 (2.2–12.5)	<0.0001

*Unless otherwise indicated; ^†^ From Wilcoxon two-sample test for difference in medians or Pearson chi-square test of independence of groups. p values <0.002 remained significant after Bonferroni's adjustment for multiple comparisons; ^‡^Hemoglobin oxygen saturation; ^§^Chronic transfusion therapy; ^||^Stage I or higher.

SBP = systolic blood pressure; DBP = diastolic blood pressure; MAP = mean arterial pressure; WBC = white blood cell count; LDH = lactate dehydrogenase; AST = aspartate aminotransferase; ALT = alanine aminotransferase; ALP = alkaline phosphatase; ACR =  urine albumin-to-creatinine ratio; eGFR = estimated glomerular filtration rate; CKD = chronic kidney disease; TRV = tricuspid regurgitant jet velocity; NT-proBNP =  N-terminal prohormone of brain natriuretic peptide.

### Elevated pulse pressure correlates with markers of hemolysis, elevated creatinine and proteinuria in HbSS patients

**Figure 1:**
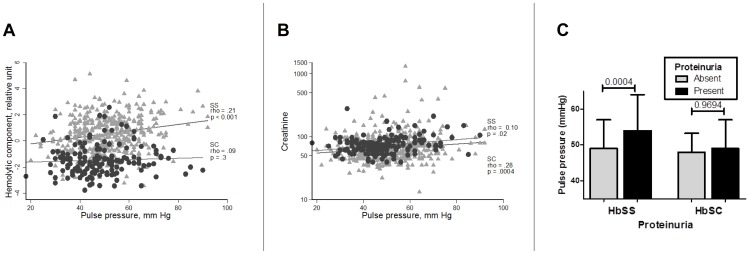
Correlates and associations of pulse pressure with kidney function and hemolysis. **A**, Pulse pressure has a significant positive correlation with the hemolytic component in HbSS patients, but not in HbSC patients. **B**, Pulse pressure has a significant positive correlation with serum creatinine in both HbSS and HbSC patients. **C**, Elevated pulse pressure is significantly associated with presence of proteinuria in HbSS patients, while the association is not significant in HbSC patients.

We next conducted a univariate analysis of pulse pressure and multiple clinical and laboratory markers in patients with HbSS and HbSC disease (Table 2). In HbSS patients, a high pulse pressure was associated with male gender and was negatively correlated with hemoglobin oxygen saturation. We also found that pulse pressure was consistently and positively correlated with all markers of hemolysis, including reticulocyte proportion, LDH, total bilirubin, AST and hemolytic component (Figure 1, panel A). Finally, pulse pressure was positively correlated with creatinine (Figure 1, panel B), urine albumin-to-creatinine ratio and presence of proteinuria (Figure 1, panel C).

**Table 2:** Correlations of pulse pressure with clinical and laboratory characteristics by hemoglobin genotype.

	HbSS patients		HbSC patients	
	n	Spearman rho (p)*	n	Spearman rho (p)*
Age	500	−0.005 (0.91)	161	0.18 (0.03)
Male gender, N(%)	500	0.15 (0.0009)	161	0.10 (0.19)
Systolic blood pressure	500	0.66 (<0.0001)	161	0.73 (<0.0001)
Diastolic blood pressure	500	−0.29 (<0.0001)	161	−0.06 (0.46)
Mean arterial pressure	500	0.09 (0.04)	161	0.33 (<0.0001)
BMI	492	−0.02 (0.64)	160	0.26 (0.001)
Hemoglobin oxygen saturation	497	−0.17 (0.0001)	160	−0.10 (0.22)
Hydroxyurea, current use, N(%)	500	−0.02 (0.70)	161	0.009 (0.91)
Chronic transfusion therapy, N(%)	499	−0.002 (0.96)	159	−0.02 (0.80)
Chronic pain, N(%)	499	−0.03 (0.47)	161	−0.06 (0.47)
Six minute walk, m	492	0.09 (0.04)	159	0.03 (0.71)
White blood cells	490	0.09 (0.04)	156	0.11 (0.15)
Hemoglobin	489	−0.06 (0.17)	157	−0.009 (0.91)
Hematocrit	490	−0.07 (0.13)	157	0.02 (0.79)
Platelets	489	0.02 (0.61)	156	0.02 (0.81)
Hemoglobin F	445	−0.01 (0.81)	139	0.02 (0.81)
Reticulocytes	461	0.16 (0.0006)	153	0.10 (0.20)
Lactate dehydrogenase	453	0.15 (0.001)	154	0.07 (0.41)
Total Bilirubin	490	0.11 (0.02)	159	0.001 (0.99)
Hemolytic Component	420	0.21 (<0.0001)	148	0.09 (0.28)
Aspartate aminotransferase	477	0.13 (0.006)	157	0.13 (0.12)
Ferritin	450	−0.05 (0.28)	141	0.22 (0.008)
Albumin	489	−0.09 (0.06)	157	−0.05 (0.54)
Alanine aminotransferase	491	0.08 (0.07)	159	0.20 (0.01)
Alkaline Phosphatase	489	0.09 (0.04)	157	0.03 (0.68)
Creatinine	492	0.10 (0.02)	159	0.28 (0.0004)
Protein in urine	474	0.16 (0.0005)	147	0.02 (0.82)
Urine album-to-creatinine ratio	306	0.15 (0.01)	81	0.20 (0.08)
Estimated glomerular filtration rate	492	−0.006 (0.9)	157	−0.25 (0.002)
Chronic kidney disease	313	0.16 (0.006)	82	0.23 (0.04)
Tricuspid regurgitant jet velocity	449	0.20 (<0.0001)	139	0.20 (0.02)
NT-proBNP†	464	0.09 (0.06)	146	0.12 (0.16)

*p values <0.002 remained significant after Bonferroni's adjustment for multiple comparisons.

† N-terminal prohormone of brain natriuretic peptide.

In HbSC patients, a different pattern of pulse pressure correlates emerged. In this group, pulse pressure was positively correlated with age and BMI. There was no correlation with markers of hemolysis and although, similarly to HbSS patients, there was a positive correlation with creatinine (Figure 1, panel B), there was no significant correlation with presence of proteinuria or the urine albumin-to-creatinine ratio.

In both HbSS and HbSC groups pulse pressure was positively correlated with TRV.

A complete list of all univariate correlations is reported in Table 2.

### Pulse pressure is the only systemic pressure parameter associated with hemolysis by multiple linear regression in HbSS patients

We conducted a multiple linear regression analysis to determine which of the variables that emerged as significant by univariate analysis were able to cross-sectionally predict the blood pressure variables (systolic, diastolic, mean arterial and pulse pressure) in the regression model. The following pairs of variables could not be entered into the model simultaneously due to their high correlation, so we fitted separate models for each of them: reticulocytes and hemolytic component; creatinine and estimated glomerular filtration rate; and proteinuria and urine albumin-to-creatinine ratio. For brevity, we are only reporting the results of the regression analysis for pulse pressure for HbSS and HbSC patients, separately.

As shown in Table 3, pulse pressure was predicted by hemolysis in HbSS patients, while systolic, diastolic and mean arterial pressure were not (data not shown).

**Table 3:** Independent predictors of pulse pressure.

	Beta (95% CI)	p	Standardized beta
**HbSS patients**			
**Model 1** (n = 387)			
Reticulocytes*, %	2.07 (0.1–4.0)	0.04	0.11
TRV, m/sec	13.03 (5.2–20.9)	0.001	0.17
Hemoglobin oxygen saturation	−0.42 (–0.79–0.05)	0.03	−0.12
Creatinine*	2.88 (0.19–5.58)	0.04	0.11
Proteinuria	2.58 (0.20–4.97)	0.03	0.11
**Model 2** (n = 352)			
Hemolytic component	1.37 (0.4–2.3)	0.006	0.16
TRV, m/sec	10.04 (1.7–18.4)	0.02	0.13
Hemoglobin oxygen saturation	−0.34 (−0.7–0.05)	0.09	−0.10
Creatinine*	3.34 (0.6–6.1)	0.02	0.13
Proteinuria	2.57 (0.08–5.0)	0.04	0.11
**Model 3** (n = 281)			
Reticulocytes*, %	2.42 (0.3–4.6)	0.03	0.13
Urine albumin-to-creatinine ratio*	0.64 (0.01–1.3)	0.05	0.12
Creatinine*	3.22 (0.2–6.2)	0.03	0.13
**Model 4** (n = 263)			
Hemolytic component	1.50 (0.4–2.6)	0.006	0.17
Urine albumin-to-creatinine ratio*	0.56 (−0.1–1.2)	0.09	0.11
Creatinine*	4.00 (0.8–7.2)	0.01	0.16
**HbSC patients**			
**Model 1** (n = 140)			
Creatinine*	9.65 (3.0–16.3)	0.005	0.23
Ferritin*	2.66 (0.7–4.6)	0.008	0.22
**Model 2** (n = 138)			
eGFR	−0.08 (−0.14, −0.02)	0.01	−0.21
Ferritin*	2.63 (0.7−4.6)	0.009	0.22

*transformed using the natural log function.

In model 1, where the hemolysis markers LDH, AST, total bilirubin, and reticulocyte proportion were coded as individual variables, the reticulocyte proportion emerged as a predictor of pulse pressure, together with TRV ≥ 3.0 m/sec, hemoglobin oxygen saturation, creatinine and proteinuria. In model 2, the hemolytic component emerged as a significant predictor of pulse pressure in the regression model. Model 3 and 4 were fitted with the urine album-to-creatinine ratio replacing proteinuria and showed similar results.

In hemoglobin SC patients, creatinine (model 1) and estimated glomerular filtration rate (model 2) and ferritin (both models) were the only predictors of pulse pressure (Table 3).

## Discussion

Pulse pressure is emerging as an important risk factor for end-organ damage and cardiovascular morbidity and mortality in many conditions (reviewed in [18]). While several studies have explored the associations of systolic blood pressure in SCD, the role of pulse pressure has never been thoroughly investigated in this disease.

Our study shows that pulse pressure is predicted by hemolysis in patients with HbSS from the walk-PHaSST international multi-center cross-sectional cohort. To our knowledge, this is the largest SCD cohort since the CSSCD, a natural history study that followed SCD patients in the pre-hydroxyurea era. A striking finding of the CSSCD was that SCD patients, and particularly those with HbSS had lower overall median systolic blood pressure (113 ± 14.5 mmHg) than normative controls, a difference observed for all age groups and both sexes.[1] In the hydroxyurea era, the UNC cohort was characterized by a higher systolic blood pressure (122 ± 15 mmHg) which was not significantly different from that of age-matched African American control subjects.[5] Both and other studies have shown that elevations of systolic blood pressure over baseline are detrimental in SCD patients and predict a risk of vascular complications including stroke,[1], [27] kidney disease,[28], [29] and pulmonary hypertension.[24] It is, however, intriguing that while high baseline hemolysis is independently correlated with these complications, no association had been found between systolic blood pressure and severity of hemolysis.[5] Our findings of a lack of association between markers of hemolysis and systolic blood pressure in multiple regression analysis in the walk-PHaSST cohort confirm these prior reports. We, therefore, hypothesized that the detrimental effect of systolic blood pressure may be mediated by pulse pressure elevation in response to hemolysis-derived vasculopathy. To this day, little attention has been devoted to pulse pressure in SCD, an increasingly recognized cardiovascular risk factor. While the mechanistic link between hemolysis, pulse pressure and vascular complications cannot be proven in an epidemiological cross-sectional study, our findings do suggest that elevated pulse pressure may be more reflective of the peculiar vasculopathy of SCD, where hemolysis causes increased arterial stiffness from nitric oxide depletion and decreased peripheral vascular resistance from anemia, leading to elevated pulse pressure. Moreover, hemolysis is also linked to microvascular dysfunction in SCD, since the primary pathogenic process in this disease occurs at the level of the post-capillary venules and is characterized by cellular adhesion, vaso-occlusion, hemolysis and ischemia-reperfusion injury.[30] Microvascular damage would lead to loss of small vessels and consequent increased arterial stiffness which would elevate pulse pressure. Elevated pulse pressure would in turn damage small vessels and lead to microvascular rarefaction in a vicious cycle.[18], [31] It is, therefore, possible that hemolysis may mechanistically link both micro and microvascular dysfunction and that elevated pulse pressure may be a result of cross-talk between large and small caliber vessels as documented in hypertensive patients.[18] The link between hemolysis and pulse pressure is also supported by our finding that hemolysis was not associated with pulse pressure in HbSC, a subgroup of SCD which is not characterized by high level hemolysis and whose disease manifestations may result, instead, from increased blood viscosity.

As other studies have shown,[5], [32] we found a strong correlation between all pressure parameters and kidney function measured as serum creatinine and proteinuria. This finding reinforces the importance of systemic blood pressure as a predictor of kidney deterioration in SCD. Since abnormalities in kidney function are among the most sensitive and earliest biomarkers of microvascular dysfunction in SCD,[33] it may be important to therapeutically target blood pressure early and aggressively, to prevent this complication. While angiotensin-converting enzyme inhibitors may reduce proteinuria in SCD patients,[34] there is no clear evidence to show which patients may benefit the most from these medications. In particular, it is not known what the optimal blood pressure threshold to initiate angiotensin-converting enzyme inhibitor therapy should be and whether reduction of albuminuria predicts long term clinical outcomes such as need for renal replacement therapy and death. Based on our results, treatments aimed at reducing hemolysis may, however, also benefit the kidney microvascular district, independently from other therapeutic pathways.

We also found a significant association between BMI and pulse pressure in HbSC patients, but not in HbSS patients. In HbSC patients, we also observed an interaction of gender and BMI, with associations of BMI with pulse pressure observed in males but not in females (data not shown). BMI has been positively correlated with pulse pressure in other populations without SCD,[35] thus suggesting that in older HbSC patients with increased BMI, obesity may have the same adverse impact on arterial compliance observed in populations exposed to routine cardiovascular risk factors. It is intriguing to hypothesize that other co-morbid conditions such as dyslipidemia and diabetes may mediate the association between BMI and pulse pressure in HbSC patients.

Finally, we found significant independent associations between hemoglobin oxygen saturation and pulse pressure in HbSS patients and ferritin and pulse pressure in HbSC patients. We hypothesize that the inverse relationship between hemoglobin oxygen saturation and pulse pressure in HbSS patients may be either the result of increased cardiopulmonary disease burden in patients with high pulse pressure, as also shown by the association with elevated TRV, or worse anemia secondary to hemolysis. As shown in Table 1, patients with HbSC had significantly lower ferritin than HbSS patients with the IQ range below 500 pmol/L. Patients with HbSC have significantly lower burden of hemosiderosis as compared to HbSS patients as they are less frequently transfused due to higher baseline hemoglobin. Thus, values in the range observed in this study may be more reflective of an enhanced inflammatory state. If this hypothesis is correct, pulse pressure may be positively correlated with increased inflammation in HbSC patients from our cohort.

The main limitation of our study is that the walk-PHaSST clinical cohort was not designed to specifically evaluate baseline systemic blood pressure in SCD patients. Thus, data on the history of hypertension or use of antihypertensive medications are not available for our population. The use of principal components analysis to derive a hemolytic component variable from several correlated variables all related to hemolysis is an additional limitation because of potentially important associations that could have been missed by using a data reduction technique such as PCA. Finally, eGFR and creatinine are relatively poor markers of kidney function in sickle cell disease due to glomerular hyperfiltration and increased creatinine excretion by the proximal tubule in this disease. It is, unfortunately, not possible to infer which patient subsets may have experienced worse hyperfiltration and the confounding effect of this phenomenon, although it is safe to assume that glomerular hyperfiltration decreased with increasing age in our population. Associations of eGFR with pulse pressure did not, however, change after adjustment for age. Our results are also strengthened by the availability of values of proteinuria and albumin to creatinine ratio in a subset of patients. These are recognized valuable screening tools of kidney dysfunction by the recently published sickle cell guidelines.[36]

In summary, our results show that pulse pressure is closely linked to hemolysis in SCD and suggest that it may be an important predictor of cardiovascular risk. Longitudinal and mechanistic studies should be conducted to confirm these hypotheses. Moreover, measurement of pulse wave velocity may provide further information on arterial stiffness and its relationship to pulse pressure and microvascular dysfunction in SCD.
